# A systematic review of the association between women’s morphological traits and fertility

**DOI:** 10.1017/ehs.2025.10026

**Published:** 2025-11-03

**Authors:** Linda H. Lidborg, Lynda G. Boothroyd

**Affiliations:** Department of Psychology, Durham University, Durham, UK

**Keywords:** sexual selection, sexual dimorphism, femininity, biological fitness, fertility, reproductive success

## Abstract

Typically feminine morphological traits in women include a neotenous facial structure with large eyes, full lips, and an oval face shape, and a curvaceous body with large breasts, a narrow waist, and full hips and buttocks. Compared to men, women also show higher second-to-fourth finger (2D:4D) ratios as well as less muscle mass, lower physical strength, and a higher voice pitch. Due to a putative association with oestrogen levels, feminine traits are often claimed to cue women’s reproductive potential. However, the evidence for this is scarce and inconsistent, typically measuring proxies rather than actual fertility outcomes. Here, we report a systematic review of direct fertility measures as a function of morphological traits in women, including breast size, waist-to-hip ratio (WHR), voice pitch, strength, and 2D:4D; no articles were found measuring facial femininity. The review included 19 articles comprising 68 effect sizes (31 samples from 16 countries; total *N* = 125,062). Our review showed that a less feminine WHR may cue past fertility, and a more feminine 2D:4D may be, at best, weakly associated with fertility. Overall, we conclude that the current evidence base is too weak to support the claim that women’s feminine morphological traits are associated with reproductive potential.

## Graphical abstract

**Figure:**
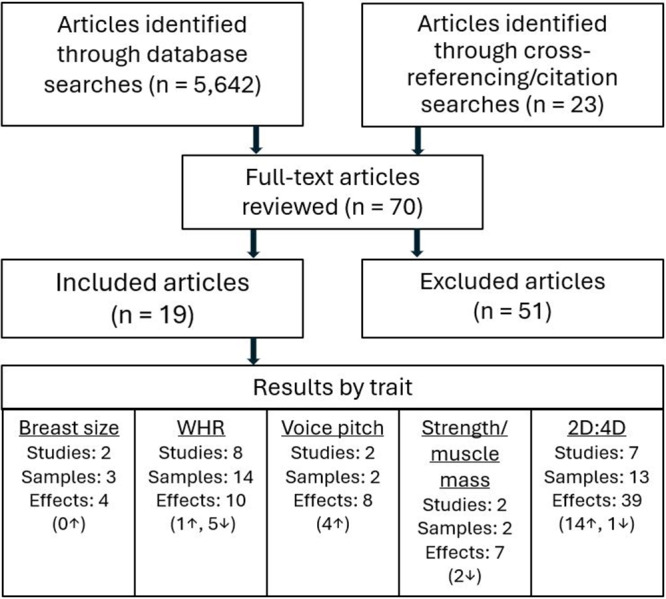

## Social Media Summary

This systematic review shows limited evidence for any association between women’s feminine traits and their fertility.

## 1. Introduction

Across both animal and plant species, males and females within the same species may differ from one another to varying degrees. Such sex differences can refer to so-called ‘primary’ differences − differences in traits related to reproduction, such as sexual organs (which may be inevitable in a sexually reproducing species) – and ‘secondary’ differences, which are unrelated to reproduction but are involved in acquiring a mate (Plavcan, 2001). The latter are referred to as ‘sexually dimorphic’ traits and they typically develop or become exaggerated at sexual maturity. Human males and females exhibit sexual dimorphism in a number of morphological traits. Compared to men, women have on average a more neotenous facial structure with a more oval face shape, a longer forehead, larger eyes, a smaller nose, a smaller chin, and fuller lips (Perrett *et al*., 1994; Rhodes, 2006). Men and women differ in both the amount and distribution of body fat: while men in most populations typically have an average body fat percentage around 10–15%, women display an average body fat percentage twice as high, ranging between 20% and 30% (Wells, 2007). Women also store body fat primarily in the gluteofemoral region; that is, on the hips, buttocks, and thighs, whereas men accumulate fat mainly in the abdominal area (Norgan, 1997). This dimorphism in body fat distribution also contributes to women of childbearing age displaying a more hourglass-shaped body with a lower waist-to-hip ratio (WHR), with ratios in women typically falling between 0.67 and 0.80 compared to between 0.85 and 0.95 in men (at least in western populations: Marti *et al*., 1991; some non-western populations of women show a relatively higher, rather than lower, WHR during childbearing years: Yu & Shepard, 1998). Adult women also have permanently enlarged breasts (Marlowe, 1998). Compared to men, women are also shorter (Gray & Wolfe, 1980), carry less muscle mass (Lassek & Gaulin, 2009), show lower strength (Lassek & Gaulin, 2009), have less facial and body hair (A. Dixson *et al*., 2005), have a higher voice pitch (Puts *et al*., 2012a), and (at least in some populations) a higher second-to-fourth finger (2D:4D) ratio (Apicella *et al*., 2016; Manning, 2002).

Some of women’s feminine morphological traits become exaggerated under the influence of oestrogen. For example, whereas male and female WHRs are very similar in childhood, girls’ increasing pubertal oestrogen levels stimulate fat deposits in the gluteofemoral area, resulting in them developing a more hourglass-shaped body (reviewed in Wells, 2007). Pubertal increases in oestrogen also influence breast growth while simultaneously hindering skeletal growth (Bordini & Rosenfield, 2011). The latter results in women having a shorter stature than men (reviewed in Dunsworth, 2020), and also leaves them with a more juvenile craniofacial structure which undergoes fewer pubertal changes compared to the changes seen in men’s faces (Rhodes *et al*., 2003). While femininity in soft facial tissues, such as lip volume, is likely also influenced by oestrogen (reviewed in Rhodes, 2006), to our knowledge this has never been demonstrated empirically. Voice pitch – which is directly related to the anatomy of the vocal folds – changes considerably less in puberty in girls than in boys, as the influence of pubertal testosterone deepens the male voice (Harries *et al*., 1998). Nevertheless, a woman’s voice pitch is under the continuous influence of oestrogen and progesterone levels throughout her life, and becomes permanently more masculinized following menopause (Abitbol *et al*., 1999). 2D:4D dimorphism, in contrast, is present already at birth (Galis *et al*., 2010) and is largely stabilized within the first two years of life (Ventura *et al*., 2012). It is often argued to reflect foetal exposure to androgens versus oestrogens (Zheng & Cohn, 2011), although this has also been questioned (Hollier *et al*., 2015; Richards *et al*., 2019). Meta-analytic evidence suggests that 2D:4D ratios are not related to adult hormone levels (Hönekopp *et al*., 2007) and they are therefore typically treated as proxies for prenatal rather than pubertal or adult hormone levels.

### 1.1. The evolution of feminine morphological traits in women

To what extent the aforementioned traits evolved and/or are maintained in women through natural or sexual selection is debated (see, e.g., Lassek & Gaulin, 2022; Motta-Mena & Puts, 2017; Pawłowski & Żelaźniewicz, 2021). A thorough discussion of how and why these selection pressures may have been exerted is beyond the scope of this article, but for clarity we make the following observations. Both types of selection pressures are likely to have influenced the evolution of female morphology in concert (Lassek & Gaulin, 2022). For example, women’s comparatively wider pelvis, which contributes to but does not fully explain their lower WHR, is necessary to allow the birth canal to be wide enough for successful childbirth. WHR is further resultant from gluteofemoral fat stores, which are used during lactation (Norgan, 1997; Rebuffé-Scrive *et al*., 1985). Evidence suggests that these fat deposits store docosahexaenoic acid, an omega-3 fatty acid which some authors have proposed to be particularly important for infant brain development (reviewed in Lassek & Gaulin, 2008, 2019). The evolution of women’s more curvaceous body shape, with a lower WHR, may thus be the result of the unique and extreme energy demands placed on human females through pregnancy and (at least ancestrally) a very extended period of lactation, pointing to natural selection pressures. An alternative – but not mutually exclusive – account posits that the evolution of bipedality promoted a lower centre of body mass in women (i.e., gluteofemoral fat deposits), with reduced shoulder width compared to men (Pawłowski & Grabarczyk, 2003), to maintain body stability during pregnancy, lactation, and when carrying young children (Pawłowski, 2001).

However, heterosexual men also tend to be sexually attracted to a low WHR in women (Dixson *et al*., 2010; Singh *et al*., 2010; Sorokowski *et al*., 2014; Thornborrow *et al*., 2018). It has therefore been suggested that women have been subjected to simultaneous sexual selection pressures for this type of body shape. It can be noted here that some evidence suggests that such preferences may not be universal (Dixson *et al*., 2007; Marlowe & Wetsman, 2001; Sugiyama, 2004; Wetsman & Marlowe, 1999; Yu & Shepard, 1998). However, this may be explained by differences in the use of 2D versus 3D (or frontal versus profile-viewed) body stimuli. Namely, when 3D or profile views are used (where the buttocks can be viewed in addition to the waist/hips), men typically show a strong preference for a relatively low WHR. There may, however, exist some geographical variation in the extent to which men prioritize wider hips or more protruding buttocks (Marlowe *et al*., 2005), both of which contribute to a lower ratio. Moreover, the most preferred WHR appears to have changed somewhat across historical periods, but has generally fallen within comparatively low values of approximately 0.68–0.74 (Bovet *et al.,* 2015).

It is similarly debated whether primarily natural or sexual selection pressures have contributed to the evolution of breasts in women. Prior to pregnancy, women’s permanently enlarged breasts are largely made up of fat tissue (Alex *et al*., 2020; Howard & Gusterson, 2000), and larger breast volume does not increase subsequent milk production capacity, nor does it facilitate breastfeeding (Caro & Sellen, 1990; Howard & Gusterson, 2000). Human breasts thus appear to mostly constitute a fat store with no obvious reproductive purpose. Human females are the only mammal to display permanently enlarged breasts from sexual maturity onwards (Cant, 1981; Wessel, 2016); other great ape females do not exhibit breasts until the end of their first pregnancy (Short, 1979). Thus, permanently enlarged breasts are clearly not necessary for lactation. Similarly to a curvaceous body shape with a low WHR, breasts are, however, sexually attractive to heterosexual men (e.g. Dixson *et al*., 2015, 2011), although this is also subject to cross-cultural variation (reviewed in Pawłowski & Żelaźniewicz, 2021). The evolution of permanent breasts in humans has therefore variously been proposed to reflect natural selection pressures for another fat deposit or sexual selection pressures resulting in breasts as a sexual ornament.

In terms of women’s feminine facial structure, there are no clear survival or reproductive benefits associated with having more feminine facial features. Unsurprisingly, the evolution of women’s facial femininity therefore tends to be explained primarily in terms of sexual − rather than natural − selection pressures. While this is supported by the observation that men are generally attracted to facial femininity in women (e.g. Fiala *et al*., 2021; Foo *et al*., 2017; Shiramizu *et al*., 2020; but see also Boothroyd *et al*., 2021; de Barra *et al*., 2013; Marcinkowska *et al*., 2014 for evidence of cross-cultural variation in men’s facial femininity preferences), there are caveats regarding this claim which are elaborated upon in the Discussion.

Research investigating sexual dimorphism in voice pitch and strength/muscle mass has typically focused more on the evolution of these exaggerated traits in men rather than in women (see e.g. Hill *et al*., 2013; Lidborg *et al*., 2022; Puts *et al*., 2006). However, a higher, more feminine voice pitch is considered attractive in a woman (Collins & Missing, 2003; Jones *et al*., 2008), possibly because it is perceived as more youthful (Puts, 2010) as the female voice becomes more masculine with age (Abitbol *et al*., 1999). A muscular physique has historically not been seen as appealing in women, although this view may be changing somewhat, at least in the west (Bozsik *et al*., 2018). This shift likely reflects changing cultural ideals and is not suggested to be related to evolutionary selection pressures.

Overall, although it is plausible that natural selection has played a major role in the evolution of at least some of the feminine traits exhibited by women (Lassek & Gaulin, 2022), the evolutionary literature nonetheless tends to be heavily skewed towards investigating these traits’ sexual appeal to men. Explanations thus tend to focus on the role of sexual selection pressures in shaping these traits (Puts, 2010; Puts *et al*., 2012b). It is pertinent to note here that in polygynous and serially monogamous populations, reproductive variance is lower in women than in men (Brown *et al*., 2009), which should have relaxed sexual selection pressures on ancestral women.

A relevant question is whether female dimorphic traits in non-human primate species are sexually or naturally selected. Non-human primate species commonly show male-biased body size dimorphism, where sexual selection pressures might strongly favour increased body size in males. In females, in contrast, it is likely that body size is under greater influence of natural selection pressures, due to size-associated differences in survival and reproductive rates (Cassini, 2023; Leigh, 1992; Plavcan, 2004). Moreover, although sexual ornaments are overall rare in female primates, females of some species (e.g., baboons, barbary and rhesus macaques, and chimpanzees) exhibit ornaments in the form of sexual swellings. These swellings indicate sexual receptivity; it has been proposed that their size signals the ‘quality’ and condition of the female (Pagel, 1994; Street *et al*., 2016) and that males are preferentially attracted to females showing larger swellings (Domb & Pagel, 2001; but see contradictory evidence reported by Deschner *et al*., 2004). These females have also been reported to show higher fertility than those with smaller swellings (Domb & Pagel, 2001; but see re-analysis of the same data by Zinner *et al*., 2002). Thus, natural and sexual selection pressures likely act in concert on dimorphic traits in non-human primate females, with the caveat that sexually selected ornaments are relatively rare. Furthermore, primate sexual swellings function to advertise the fertile period; human females have concealed ovulation, and the traits reviewed here in humans are permanent traits.

### 1.2. Do feminine morphological traits cue reproductive potential?

Pubertal oestrogen exposure thus modulates women’s feminine trait development, with some traits becoming exaggerated as a result of rising oestrogen levels (i.e. body shape) and other traits ceasing to develop when oestrogen levels increase (i.e. craniofacial structure). It is sometimes claimed that the relationship between femininity and oestrogen continues beyond adolescence, so that feminine trait expression in adulthood is also correlated with adult oestrogen levels. Evidence for a link between women’s morphological traits and adult hormone levels is mixed, however. Positive relationships with oestrogen levels have been reported for facial femininity (Law Smith *et al*., 2006), facial (Durante & Li, 2009; Law Smith *et al*., 2006; Żelaźniewicz *et al*., 2020) and bodily attractiveness (Durante & Li, 2009; Grillot *et al*., 2014), a more feminine body shape (Jasieńska *et al*., 2004) and right-hand but not left-hand 2D:4D (McIntyre *et al*., 2007). Other researchers, however, have observed no associations between women’s oestrogen levels and their body shape (Grillot *et al*., 2014; Jones *et al*., 2018; Rilling *et al*., 2009) or their attractiveness (Jones *et al*., 2018; Rilling *et al*., 2009). Furthermore, associations with testosterone have sometimes been observed: facially attractive women may have lower testosterone levels overall or a low testosterone-to-oestradiol ratio (Probst *et al*., 2016; Wheatley *et al*., 2014; Żelaźniewicz *et al*., 2020). Similarly, van Anders and Hampson (2005) reported a weak but significant positive relationship between women’s WHR and testosterone levels. However, Mondragón-Ceballos *et al.* (2015) found that women with the lowest WHRs had high levels of both testosterone and oestradiol, but this was only the case for women in the fertile phase of the menstrual cycle. Null relationships between testosterone levels and body shape/body attractiveness have also been reported (Grillot *et al*., 2014). Indirect evidence that oestrogen plays a role in the maintenance of feminine trait expression is seen in that women’s soft facial tissues and their body shape become masculinized when menopause is reached and hormone profiles change, with a decrease in oestrogens and an increase in testosterone (Furnham *et al*., 1997; Howard & Gusterson, 2000). As a general caveat, it should be noted here that sample sizes in the cited hormone literature are generally small, with *N* ranging from 33 to 249 in the studies cited above (median *N* = 56). It is therefore important not to overinterpret nonsignificant results in studies that are, in some cases, likely to lack the power to detect weak-to-moderate effect sizes. Simultaneously, however, this also raises concerns over the replicability of some significant, strong effects that have emerged in some of the smaller samples (Funder & Ozer, 2019) and not been repeated in other studies. Nonetheless, the argument that feminine facial and body traits reflect adult oestrogen levels persists in the literature, with the absence of consistent and direct evidence for such a relationship rarely being acknowledged.

Because oestrogens, such as oestradiol, play a role in women’s fertility (Lipson & Ellison, 1996; Venners *et al*., 2006), feminine traits are commonly posited to either directly signal or indirectly cue women’s reproductive potential (reviewed in Weeden & Sabini, 2005). In short, due to the (putative) link between femininity and oestrogen, women with more feminine traits are expected to have higher fecundability at a given timepoint (i.e. greater probability of conception), higher fecundity (i.e. greater reproductive potential), and higher fertility overall (i.e. more children over their lifetime). Via inclusive fitness, higher fertility may also include improved offspring condition and survival. Under this hypothesis, men should gain reproductive benefits by mating with more feminine women as this should increase their own reproductive output. Accordingly, men should have evolved preferences for traits that cue fecundity (or, in some cases, fecundability), and they should therefore exhibit preferences for feminine traits in women. This central tenet forms the core of sexual selection explanations for the evolution of women’s dimorphism. In support of this (and as already discussed), traits such as a feminine facial structure and a WHR in the lower end of the typical range are often found to be regarded as attractive by heterosexual men (Dixson *et al*., 2010; Fiala *et al*., 2021; Thornborrow *et al*., 2018, but see also, e.g. Boothroyd *et al*., 2021; Scott *et al*., 2014 for cross-cultural evidence suggesting that femininity is not always preferred).

In summary, although the hypothesis that women’s femininity cues their reproductive potential appears to make logical sense, for it to be supported, certain conditions must be met. First, feminine traits should reliably index oestrogen levels – which we have seen is not necessarily the case. Second, heterosexual men should be attracted to more exaggerated feminine traits in women. The evidence for this notion is stronger, although cross-cultural evidence indicates that (at least for some traits) this may not be completely universal. Third, women with more feminine traits should show greater fertility, at least under natural fertility conditions. (It is pertinent to note here that, if natural – rather than sexual – selection is the primary driver of women’s morphology, we would *still* expect morphological traits to be associated with greater fertility.) Despite the fact that the latter is an extremely widespread claim in the evolutionary literature, empirical support for this claim is scarce.

Evidence for the third condition – that women’s facial and bodily traits cue their fertility – typically relies heavily on fertility proxies, for which oestrogen levels (outlined above) is one example. Third-party ratings are also sometimes used as ‘evidence’, whereby (typically western) participants rate photographs or line drawings of more feminine and attractive women as being more fecund or fertile (e.g. Andrews *et al*., 2017; Furnham *et al*., 2001; Singh, 1993). This is problematic: to our knowledge, no research exists demonstrating that third-party raters can accurately determine how fertile women are simply based on photographs. There is also evidence suggesting that such perceptions may not be present in non-industrialized populations (Sorokowski *et al*., 2014), indicating that cultural influences might play a role. People (and this applies to researchers as well) are subject to numerous biases in face and body perception. It is certainly possible that research participants’ perception that femininity and attractiveness are associated with fertility may simply be yet another example of the attractiveness halo effect, whereby more attractive people are assumed to possess a range of favourable attributes regardless of whether that is actually true (Dion *et al*., 1972; Langlois *et al*., 2000).

Other proxies for fertility are also used, oftentimes measured as a function of women’s WHR. For example, women with a lower, more feminine WHR might produce children in better condition at birth, at least according to some studies (reviewed in Bovet, 2019; see also Lassek & Gaulin, 2018). Women attending fertility clinics (i.e. women who experience difficulties getting pregnant) may be more likely to conceive if they have a lower WHR (Zaadstra *et al*., 1993). Furthermore, women with a higher, less feminine WHR have been found to take longer to get pregnant (Sundaram *et al*., 2017). To reiterate, this constitutes indirect rather than direct evidence, and these findings do not actually demonstrate that more feminine women produce more children over their lifetime. The use of fertility proxies is potentially problematic: we recently published a meta-analysis where we observed that some masculine traits in men (e.g. increased height) positively predicted mating success (largely measured in industrialized populations), which is a commonly used proxy for reproductive success in men. However, contrary to expectations, those same traits were not associated with men’s reproductive success in natural fertility contexts (Lidborg *et al*., 2022). This demonstrates the potential perils of relying too heavily on fertility proxies. Moreover, the same meta-analysis showed that several of men’s masculine traits (e.g. facial masculinity) were not actually associated with reproductive success at all, despite widespread claims that they should be. Our meta-analytic results in men thus illustrate the need to test actual reproductive outcomes rather than fertility proxies. Further, they also caution against the common tendency to extrapolate findings across cultural contexts which might vary considerably in life-history profiles and contraceptive use. When it comes to women, WHR studies suffer from the additional caveat that any findings showing that a lower WHR predicts greater fertility may only present in well-nourished industrialised contexts where obesity levels are high, as WHR is positively correlated with BMI – and obesity is highly unlikely to have existed ancestrally (Eaton *et al*., 1997). Overall, this means that perhaps the most common claim regarding the evolution of morphological traits in human females rests upon a shaky ground of assumptions rather than actual evidence.

### 1.3. The present article

Here, we focus on the third condition described above: that women’s feminine morphological traits are associated with greater fertility. To the extent that this has been tested, researchers have – as we have seen − commonly relied on proxies for fertility rather than measuring actual fertility outcomes. In the present article, we therefore conducted a systematic literature review of actual fertility as a function of women’s feminine morphological traits. To our knowledge, this has never been done previously. We emphasize that our aim was not to attempt to determine whether natural or sexual selection pressures have been more influential in the evolution of female morphology; our aim was merely to test whether this putative association (which is more commonly observed as an argument in the sexual selection literature) exists in the available evidence base. Our search of data included measures of facial femininity, breast size, WHR, voice pitch, and 2D:4D. Our previous meta-analysis of men’s masculine traits showed that men’s physical strength and muscularity positively predicted reproductive outcomes. To rule out the possibility that such a relationship is mediated by maternal strength/muscularity, in this review we also included these traits which are typically exaggerated in men.

## 2. Methods

### 2.1. Literature search and study selection

**Figure 1.**
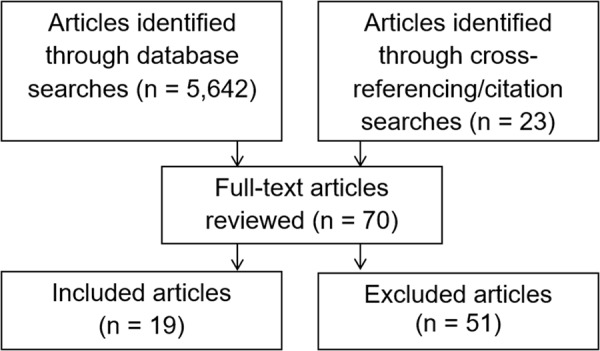
Overview of the systematic literature search.

A systematic literature search was initially conducted in August 2020 and updated in October and November 2024 on the databases PubMed, PsycINFO, and Web of Science. The following search terms were used: (feminin* OR ‘face shape’ OR ‘facial shape’ OR dimorphism OR dimorphic OR morpholog* OR width-to-height OR fWHR OR ‘breast size’ OR ‘breast volume’ OR ‘waist-to-hip ratio’ OR WHR OR waist OR ‘digit ratio’ OR 2d:4d OR ‘voice pitch’ OR ‘vocal pitch’ OR voice OR ‘fundamental frequency’) AND (‘number of offspring’ OR ‘offspring number’ OR ‘number of children’ OR ‘number of grandoffspring’ OR ‘number of grand offspring’ OR ‘offspring health’ OR ‘offspring mortality’ OR interbirth OR ‘mortality of offspring’ OR ‘surviving offspring’ OR ‘offspring survival’ OR ‘reproductive onset’ OR ‘reproductive success’ OR ‘first birth’ OR fertil* OR fecund* OR ‘biological fitness’ OR ‘Darwinian fitness’) AND (human OR woman OR women OR participant*) NOT cancer NOT embryo* NOT blastocyst NOT sperm NOT kisspeptin. Search alerts were set up on Google scholar to ensure that later publications would be found. Citation searches of all eligible articles were also conducted on Google scholar to allow us to identify unpublished data in, for example, theses and dissertations. Articles were further found through cross-referencing, and requests for published and unpublished data were made on social media. No unpublished data were identified. As Fig. 1 shows, a total of 5,642 systematic search results, including duplicates, were retrieved. An additional 23 articles were found through other methods. Based on article titles, 152 articles were selected to have abstracts scanned; of these, 70 articles were read in full.

For inclusion in the review, we considered articles that had analysed at least one of the following morphological traits: facial femininity, breast size, WHR, voice pitch, and 2D:4D. As previously explained, we also included strength and muscle mass as predictors. Most work on fertility as a function of female morphology has assessed fertility in relation to height. This relationship is strongly influenced by nutritional status (Silventoinen, 2003) rather than reflecting sexually dimorphic morphological traits, which is the main focus of this article. The literature on women’s height predicting fertility is therefore not reviewed here. With the exception of height, this systematic review thereby largely mimics our previous meta-analysis in men (Lidborg *et al*., 2022).

To be included in the review, articles also had to have analysed at least one of the following fertility measures: number of children/grandchildren (born or living), child survival/mortality, number of pregnancies, age at first birth, age at last birth, interbirth interval, and reproductive lifespan. These measures thus encompass both absolute fertility and offspring survival, as well as downstream reproductive success via grandchildren, capturing offspring condition indirectly through inclusive fitness. Only articles written in a language the first author is fluent in (English and Swedish) were considered. Studies had to have analysed men and women separately. Three articles were located that included relevant variables but did not report the relevant associations; the authors of those articles were contacted but as they were not able to provide the effect sizes, those articles were excluded. We were not able to locate any unpublished studies. In total, 19 articles were eligible for inclusion in the review, comprising 68 effect sizes from 31 samples in 16 countries and one worldwide online sample (total *N* = 125,062 unique participants). Although best practice would be a longitudinal design, where morphological traits are measured prospectively in nulliparous women with total fertility later measured retrospectively, all articles in the review bar one measured the associations cross-sectionally in samples that often included women from a large age range across pre-, mid- and post-reproduction stages of life. This affects the interpretation of the observed relationships, as some traits (particularly breast size and WHR) are influenced by reproduction. The results of such studies should therefore be interpreted with caution.

As can be seen in Table 1, two articles were found reporting associations for breast size, eight for WHR, two for voice pitch, two for strength, and seven for 2D:4D. Despite our best efforts, we were not able to locate even a single article reporting the association between facial femininity and fertility. Thus, the small number of studies for some traits prevented a formal meta-analysis. For WHR and 2D:4D, a meta-analysis was technically possible, but heterogeneity in measures would have required us to exclude some of the studies. Combined with the small number of studies, this would have risked biasing the results, and we therefore opted for a narrative review.

**Table 1.** Summary of associations between women’s morphological traits and fertility measures

Author(s) and year	Sample and age of participants	Morphological trait	Fertility measure	*N*	Effect
Breast size					
Manning *et al*., 1997	Healthy attendees at the University of Liverpool Breast Screening Unit, UK Age: 27−65	Breast volume	No. of children	500	Null
					*F* = 0.024, *p* = 0.88
			Age at first birth	500	Null
					*F* = 0.029, *p =* 0.87
Møller *et al*., 1995	Women who had been in contact with a medical doctor, Spain Age: 16−73	Breast size	No. of children	172	NR
					β = 0.10, *p =* NR
	Women who had attended a consultation for breast plastic surgery, US Age: 14−68	Breast size	No. of children	50	NR
					β = −0.21, *p* = NR
WHR					
Butovskaya *et al*., 2017	Hadza, Datoga, and Isanzu, Tanzania; Ob Ugric people, Russia; Minahasans and Sangirese, Indonesia; Tsimane’, Bolivia Age: 13−95	WHR	No. of children	925	+ve
					*B* = 0.154, *p* < 0.001
Manning *et al*., 2000	Community sample, UK	WHR	No. of children	95	Null
	Age: *M* = 56				*B* = −0.85, *p* = 0.65
Lassek & Gaulin, 2006	Third National Health and Nutrition Examination Survey (NHANES III), US Age: 11−49	WHR	Parity	3,917	+ve
					*B =* 0.007, *p* < 0.001
Nenko & Jasienska, 2009	Mogielica Human Ecology Study Site, Poland (same as Nenko & Jasienska, 2009)	WHR	Low (≤3 children) vs high parity (>3 children)	276	Null
					*F*_1,267_ = 0.16, *p* = 0.69
	Age: 22−85				
Rodrigues & Da Costa, 2001	Healthy attendees at medical clinics and public health centres; university students and staff, Brazil	WHR	Parity	203	+ve
					*F* = 16.09, *p* = 0.0001
	Age: 18−43				
D. E. Smith *et al*., 1994	The Coronary Artery Risk Development in Young Adults (CARDIA) study, US Age: 18−30	WHR (at baseline)	Parity group (at follow-up): nulliparous, primiparous, multiparous	448 Black women	Null
					*M (SD)* WHR (at baseline): Nulliparous: 0.73 (0.06)
					Primiparous: 0.73 (0.05)
					Multiparous: 0.74 (0.04)
					*p* = 0.51
				680 White women	Null
					*M (SD)* WHR (at baseline): Nulliparous: 0.72 (0.05)
					Primiparous: 0.72 (0.04)
					Multiparous: 0.73 (0.05)
					*p* = 0.38
Troisi *et al*., 1995	The Nurses’ Health Study, USA Age: 40−65	WHR	Parity	44,487	+ve
					ρ = 0.10, *p* < 0.05
			Age at first birth	44,487	+ve
					ρ = 0.05, *p* < 0.05
Wells *et al*., 2010	The SizeUK survey study, UK Age: 16−40	WHR increment	Parity group: multiparous vs nulliparous	2,316	+ve
					*d* ≈ 0.089, *p =* 0.002
Voice pitch					
Apicella *et al*., 2007	Hadza, Tanzania (same as K. M. Smith *et al*., 2017) Age: 18−53	Voice pitch	No. of children born	48	Null
					β = −0.111, *p* = 0.375
			Reproductive success	48	Null
					β = −0.058, *p* = 0.678
			No. of children died	48	Null
					β = 0.023, *p* = 0.869
			Mortality rate	48	Null
					β = 0.215, *p* = 0.148
Atkinson *et al*., 2012	Himba (Ovahimba), Namibia Age: 18−80	Voice pitch	Genetic vector*	54	+ve
			One-tailed		β = 0.292, *p* < 0.01
			No. of living children One-tailed	54	+ve
					β = 0.245, *p* < 0.01
			No. of living grand-children One-tailed	54	+ve
					β = 0.239, *p* < 0.01
			No. of living grand-children (controlling for children)	54	+ve
					β = 0.168, *p* < 0.05
			One-tailed		
Strength/muscle mass					
Atkinson *et al*., 2012	Himba (Ovahimba), Namibia Age: 18−80	Handgrip strength	Genetic vector* One-tailed	54	+ve
					β = 0.281, *p* < 0.05
			No. of living children One-tailed	54	+ve
					β = 0.327, *p* < 0.01
			No. of living grand-children One-tailed	54	Null
					Effect size = NR, *p* = NR
			No. of living grand-children (controlling for children) One-tailed	54	Null Effect size = NR, *p* = NR
K. M. Smith *et al*., 2017	Hadza, Tanzania (same as Apicella *et al*., 2007)	Upper-body strength/muscle mass	Reproductive success	48	Null
					*r* = 0.18, *p =* NR
	Age: 18−53		Fertility	48	Null
					*r* = 0.09, *p =* NR
			Offspring mortality	45	Null
					*r* = −0.18, *p =* NR
2D:4D					
Helle, 2010	Community sample, Finland (born 1946−58)	2D:4D	Probability of having at least one child in lifetime	265	Null
	Age: ∼50, 55, and 60				χ^2^ = 2.48, *p* = 0.29
		R2D:4D	Age at first reproduction (only women who had reproduced)	240	Null
					*r* = −0.06, *p =* NR
			Age at last reproduction (only women who had reproduced)	240	Null
					*r* = −0.01, *p =* NR
			Mean interbirth interval (only women who had reproduced)	240	Null
					*r* = 0.07, *p =* NR
			No. of adult children (only women who had reproduced)	240	Null
					*r* = 0.01, *p =* NR
		L2D:4D	Age at first reproduction (only women who had reproduced)	240	Null
					*r* = 0.02, *p =* NR
			Age at last reproduction (only women who had reproduced)	240	Null
					*r* = 0.06, *p =* NR
			Mean interbirth interval (only women who had reproduced)	240	Null
					*r* = 0.04, *p =* NR
			No. of adult children (only women who had reproduced)	240	Null
					*r* = 0.04, *p =* NR
Klimek *et al*., 2016	Mogielica Human Ecology Study Site, Poland (same as Nenko & Jasienska, 2009)	R2D:4D	No. of children	298	+ve
					χ^2^ = 13.1, *p* = 0.003
	Age: 46−92		Age at first birth	291	Null
					*F* = 1.52, *p* = 0.22
			Age at last birth	292	Null
					*F* = 1.22, *p* = 0.27
			Reproductive lifespan	287	+ve
					*F* = 4.30, *p* = 0.04
			Mean interbirth interval	274	Null
					*F* = 0.75, *p* = 0.38
		L2D:4D	No. of children	301	Null
					χ^2^ = 1.63, *p* = 0.20
			Age at first birth	291	Null
					*F* = 0.20, *p* = 0.65
			Age at last birth	292	+ve
					*F* = 5.28, *p* = 0.02
			Reproductive lifespan	287	Null
					*F* = 2.00, *p* = 0.15
			Mean interbirth interval	274	Null
					*F* = 0.00, *p* = 0.98
Manning *et al*., 2000	Community and student sample, UK	R2D:4D	No. of children	183	+ve
	Age: ≥30		One-tailed		*B* = 5.33, *p* = 0.01
		L2D:4D	No. of children	183	+ve
			One-tailed		*B* = 6.71, *p* = 0.005
	Community sample and university staff, Germany	R2D:4D	No. of children	96	+ve
	Age: ≥30		One-tailed		*B* = 6.91, *p* = 0.01
		L2D:4D	No. of children	96	+ve
			One-tailed		*B* = 7.10, *p* = 0.01
	Community sample, Hungary	R2D:4D	No. of children	39	+ve
	Age: ≥30		One-tailed		*B* = 11.45, *p* = 0.03
		L2D:4D	No. of children	39	+ve
			One-tailed		*B* = 10.04, *p* = 0.035
	Community sample, Poland	R2D:4D	No. of children	103	Null
	Age: ≥30		One-tailed		*B* = −4.93, *p* = 0.46
		L2D:4D	No. of children	103	Null
			One-tailed		*B* = −2.29, *p* = 0.63
	Community sample, Jamaica	R2D:4D	No. of children	60	Null
	Age: ≥30		One-tailed		*B* = 6.88, *p* = 0.15
		L2D:4D	No. of children	60	Null
			One-tailed		*B* = 9.32, *p =* 0.11
Manning *et al*., 2003	Community sample, UK	2D:4D	No. of surviving children	214	+ve
	Age: ≥23				*r* = 0.18, *p* = 0.01
	Sugali and Yanadi tribal groups, India	2D:4D	No. of surviving children	80	Null
	Age: ≥18				*r* = 0.21, *p* = 0.07
	Zulus from townships near Durban, South Africa	2D:4D	No. of surviving children	132	−ve
	Age: ≥40				*r* = −0.25, *p* = 0.04
Manning & Fink, 2008	Online sample, worldwide	R2D:4D	No. of children	69,173	+ve
	Age: ≥18				*r* = 0.018, *p* = 0.0001
			Age at first child	22,369	−ve
					*r* = −0.039, *p* = 0.0001
		L2D:4D	No. of children	69,173	+ve
					*r* = 0.010, *p* = 0.002
			Age at first child	22,369	−ve
					*r* = −0.026, *p* = 0.0001
Marczak *et al*., 2018	Yali people of Papua, Indonesia	R2D:4D	No. of children	32	Null
	Age: 20−56				*F* = 0.78, *p* = 0.38
		L2D:4D	No. of children	32	Null
					*F* = 0.76, *p* = 0.39
Vehmas *et al*., 2006	Teachers and dentists, Finland	2D:4D	No. of pregnancies	489	Null
	Age: 45−63				*r* = 0.006, *p* = 0.942

* Note. *1 × (number of living children) + 1/2 × (number of living grandchildren); NR = not reported; L2D:4D = left 2D:4D; R2D:4D = right 2D:4D; WHR = waist-to-hip ratio; +ve = positive effect; −ve = negative effect. As the predicted direction of effects differs between studies depending on measures used, effects showing significantly greater fertility as a function of *increased* femininity are indicated in 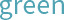 and as a function of *decreased* femininity in 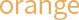.

## 3. Results

For clarity, if predictions derived from sexual selection-based explanations of women’s feminine morphological traits are correct, more feminine women should have greater fertility, indexed here by: producing more children and grandchildren, having experienced a higher number of pregnancies, having a greater proportion of surviving children (and thus lower child mortality), being younger at the birth of the first child, being older at the birth of their last child (and thus having a longer reproductive lifespan), and showing a shorter interbirth interval. The predicted direction of effects, based on claims in the sexual selection literature, are stated for each trait below.

*Breast size.* Prediction: women with larger breasts should have greater fertility. However, having children also affects breast appearance (Pisacane *et al*., 2004), meaning that associations may differ depending on whether breast size was measured prospectively or retrospectively.

The two articles that were located here, comprising three industrialized samples, showed conflicting findings. Manning *et al.* (1997) observed no relationships between breast volume, assessed from mammograms, and either number of children or age at first birth in UK women. In a Spanish sample, where breast size was measured in person by a medical doctor, Møller *et al.* (1995) found that breast size was weakly and positively associated with number of children (β = 0.10; no *p* value given). However, in a US sample where breast size was measured from photographs, the same authors reported that breast size was *negatively* predictive of number of children (β = − 0.21; no *p* value given). Based on this evidence, it is therefore not possible to draw any conclusions about an association between breast size and fertility.

*WHR.* Prediction: women with a low WHR, with a narrow waist and round hips, should have greater fertility. However, similarly to breast size, women’s WHR is also affected by having children; relationships may therefore reverse from pre- to post-reproduction.

Across eight studies sampling both industrialized and non-industrialized populations, the majority of significant associations (with effect sizes ranging from very weak to strong) showed that it was a *greater* – not a smaller – WHR that was associated with greater fertility. In women from seven non-industrialized societies in Tanzania, Russia, Indonesia, and Bolivia, Butovskaya *et al.* (2017) found that WHR weakly and positively predicted number of children (*B* = 0.154, *p* < 0.001). Lassek and Gaulin (2006) also observed a positive association between WHR and parity (i.e. number of births) in a very large US sample, but the effect size was so weak it cannot be considered meaningful (*B* = 0.007, *p* < 0.001). Wells *et al.* (2010) found that multiparous UK women showed a greater increment in WHR than did nulliparous women (approximate effect size calculated from information provided in the article: *d* ≈ 0.089; *p* = 0.002), supporting the notion that childbirth leads to a higher WHR. Similarly, a strong positive association between WHR and parity was observed by Rodrigues and Da Costa (2001) in urban Brazilian women (*F* = 16.09, *p* = 0.0001). In addition, Troisi *et al.* (1995) also reported that a greater WHR weakly correlated with parity (Spearman’s rho [ρ] = 0.10, *p* < 0.05) in a very large, primarily post-reproductive US sample of nurses. Conversely, these authors also found that a greater WHR significantly predicted a higher age at first birth (ρ = 0.05 *p* < 0.0); the latter is the only significant association we observed for WHR that was in the predicted direction. However, in the only located study that used a prospective design, Smith *et al.* (1994) reported that WHR at baseline did not significantly predict parity group membership (nulliparous, primiparous, or multiparous) at a 5-year follow-up in US women. Lastly, no significant relationships were reported between WHR and number of children in either a post-reproductive UK community sample (Manning *et al*., 2000) or a cross-sectional natural fertility Polish sample (Nenko & Jasienska, 2009). Thus, this review found no consistent significant associations between WHR and fertility. However, it can be noted that the majority of associations were positive. This suggests that although we cannot conclude that nulliparous women with a lower WHR will produce more children in their lifetime, judging by the direction of effects here, it is plausible that childbirth weakly increases WHR in parous women.

*Voice pitch.* Prediction: women with a higher voice pitch should have greater fertility.

Two studies had tested the association between voice pitch and fertility, both in fairly small, non-industrialised samples. In support of the aforementioned prediction, Atkinson *et al.* (2012) reported that Namibian Himba women with a higher voice pitch had moderately greater fertility (genetic vector [calculated as 1× (number of living children) + ½ × (number of living grandchildren)]: β = 0.292, *p* < 0.01; number of living children: β = 0.24, *p* < 0.01; number of living grandchildren: β = 0.239, *p* < 0.01; number of living grandchildren (controlling for children): β = 0.168 *p* < 0.05). In contrast, Apicella *et al.* (2007) observed no significant associations between voice pitch and fertility in Tanzanian Hadza women. Thus, based on the available evidence, we cannot conclude whether voice pitch is related to fertility.

*Strength/muscle mass.* Prediction: strength and muscle mass are traits that are exaggerated in men, not in women. However, as those traits positively predict reproductive success in men, they were − as previously explained – included here to explore a potential mediating effect of maternal traits on the association observed in men. However, if strength and muscle mass are not under selection in women, strength/muscle mass should show no association with fertility.

Two studies reported the relationship between strength/muscle mass and fertility in women. In the aforementioned sample of Himba women, Atkinson *et al.* (2012) found that stronger women had a moderately greater genetic vector and more living children (β = 0.28, *p* < 0.05 and β = 0.327, *p* < 0.01, respectively) but not significantly more grandchildren. In contrast, Smith *et al.* (2017) observed no significant effects in the previously mentioned Hadza sample (the sample for which Apicella *et al*., 2007, reported voice pitch), although it should be noted that the sample size here was small and that the effects were in the same direction as the significant effects reported by Atkinson and colleagues in the Himba.

*2D:4D.* Prediction: women with a higher 2D:4D should have greater fertility.

Seven studies had analysed the association between 2D:4D and fertility, comprising 39 effects from 13 samples. Fourteen of these effects were significant and in the predicted direction; one significant effect was in the opposite direction, with the remaining 24 effects being nonsignificant. Manning *et al.* (2000) reported that women with a higher 2D:4D (researcher-measured either directly on the participants’ hands or from hand photocopies) had more children in UK, German, and Hungarian samples, but no significant effects were found in Polish or Jamaican samples. The same laboratory also found that, in a different UK sample, women with a higher 2D:4D (measured directly by a researcher) reported more children, but South African Zulu women showed a significant effect in the opposite direction, and the association was nonsignificant in an Indian sample (Manning *et al*., 2003). In a worldwide online sample, Manning and Fink (2008) also reported significant relationships between both self-reported right- and left-hand 2D:4D with number of children and age at first child, but the statistical significance of these effects appeared to result mainly from extremely large sample sizes as effect sizes ranged around zero (*r* ranging between −0.039 and 0.018; *p* ranging between 0.0001 and 0.002). Klimek *et al.* (2016) also reported that a higher researcher-measured right-hand 2D:4D predicted a limited number of higher fertility measures in a largely non-contracepting sample of post-reproductive rural Polish women, but the majority of their effects were nonsignificant. In the largely non-contracepting Yali people of Papua, Indonesia, Marczak *et al.* (2018) found no significant associations between 2D:4D (researcher-measured on the hand and from photographs) and number of children. In addition, Vehmas *et al.* (2006; 2D:4D measured from hand X-rays) and Helle (2010; hand scan measurements) reported no significant effects for 2D:4D in two samples of post-reproductive Finnish women. Overall, these results suggest that a higher, more feminine 2D:4D may at best be weakly associated with fertility.

## 4. Discussion

In the evolutionary literature, women’s feminine morphological traits (such as facial femininity, large breasts, a low WHR, and a high voice pitch) are commonly argued to cue fertility. Men are claimed to have evolved preferences for such traits because that would focus their mating efforts on women more likely to increase their own reproductive output. If this suggestion is true, it should be possible to evidence (at least in non-contracepting populations) a positive association between women’s morphology and their fertility. Furthermore, such an association could also be predicted to arise from natural rather than sexual selection pressures, although this is more seldom discussed. Establishing such an association is therefore important to further our understanding of how those traits may have evolved and/or how they are maintained in contemporary women.

Here, we systematically reviewed extant literature for associations between women’s morphological traits (breast size, WHR, voice pitch, strength/muscle mass, and 2D:4D) and fertility. Overall, we found few studies assessing these relationships. The vast majority of studies we identified had assessed fertility as a function of 2D:4D and WHR, where the results for 2D:4D showed a mixture of significant and nonsignificant effects. Most of the significant associations were in the predicted direction (i.e. a higher, more feminine 2D:4D was associated with greater fertility). However, some of the effect sizes were so small it can be questioned whether they are truly meaningful. What is to be considered a meaningful effect? Funder and Ozer (2019) discussed how even small effects cumulate over time and thus can become consequential in the long run. Small effects should therefore not automatically be dismissed. These authors suggested that correlation coefficients around 0.05–0.10 are small but potentially meaningful; some of the significant effects for 2D:4D here were below this benchmark. For example, the significant effect sizes in Manning and Fink (2008) ranged between *r* = 0.010 and 0.039.

Overall, however, the present results suggest that 2D:4D ratio – which is often claimed to reflect prenatal hormone exposure (Manning, 2002; although this is not a universally accepted claim: e.g., Richards *et al.,* 2019) – might weakly cue fertility in women. Given that this finding is based on few (and primarily industrialized) samples, however, and that several of the significant observations were from the same laboratory, future research will have to show whether this association is robust. It is also relevant to note here that 2D:4D is typically not argued to be a sexually selected trait as such; men do not tend to choose romantic and sexual partners based on their 2D:4D ratio, and, unlike typical secondary sexual characteristics, it also does not become exaggerated at puberty. We therefore consider it a possibility that the observed (weak) association between 2D:4D ratio and fertility is a spurious one.

In terms of WHR, only one study – of eight – had measured WHR prospectively; this showed no significant differences between women’s WHR in relation to parity assessed five years later. Our review therefore cannot determine whether, among nulliparous women, those with the lowest WHRs will show greater lifetime fertility than those with relatively higher WHRs; this critical question remains open. In the remaining studies (including a mix of industrialized and small-scale, non-contracepting societies), retrospectively measured WHR was in most cases associated (although not always significantly) with greater fertility, suggesting that WHR might cue past fertility (as also discussed in Bovet, 2019) at least in premenopausal women. Combined with observations that men are (generally) attracted to a low WHR, these findings could possibly be seen as consistent with the so-called ‘nubility hypothesis’ (Lassek & Gaulin, 2019), whereby men’s preferences for a low WHR in women are suggested not to stem from smaller-waisted women being more fecund. Rather, this supposedly reflects preferences for nubile women: young, sexually mature women who have not yet started reproducing. (It is, of course, also possible that a lower WHR is attractive simply because it is a cue to biological sex and/or sexual maturity; see Bovet, 2019, for a discussion of these and other hypotheses). However, it is important to note that the lack of studies assessing pre-reproductive WHR is a major limitation of the literature as a whole.

Both for breast size and voice pitch (two studies each; breast size measured in three industrialised samples and voice pitch measured in the non-contracepting Hadza of Tanzania and Himba of Namibia), the studies showed contradicting findings, preventing us from drawing any conclusions about these relationships. As discussed earlier, we chose to also include physical strength/muscle mass in our review, despite strength/muscle mass being exaggerated in men rather than women (Lassek & Gaulin, 2009). Here, we found that these traits were positively correlated with fertility in both Hadza and Himba women, although not all effects were significant. If this should hold true in other samples, this is interesting because these traits are not typically argued to be under selection in women. Furthermore, strength and muscle mass were the only traits we previously found were associated with higher reproductive success in men, and the effect sizes observed here were similar in magnitude to those observed in men. Again, however, two studies are not sufficient to draw any firm conclusions from, so it is important not to overstate the importance of the effects reviewed here.

A caveat of this review (and the literature as a whole) is that some of the samples were very small (*N* ∼ 40 in the smallest studies) and may have been underpowered to detect small to medium-sized relationships. Our recent meta-analysis in men (Lidborg *et al*., 2022) showed that the robust associations between men’s body strength/muscle mass and reproductive outcomes showed correlation coefficients of approximately 0.15; an effect size of that magnitude would require a sample size of 172 participants to be significant. Here, 43% of the effects were observed in samples smaller than that, and (assuming that any relationships between women’s morphological traits and reproductive outcomes are similar in strength to men’s) it cannot be ruled out that some of the nonsignificant findings we reviewed here might have been significant with greater power.

Moreover, it is important to note that any associations between morphological traits and reproductive outcomes that are observed in a specific contemporary human population do not necessarily reflect what those associations might have looked like ancestrally. Indeed, failure to test hypotheses in current high-fertility contexts is a weakness of the extant literature. Widespread access to contraception, voluntary childlessness, reduced mortality, and other cultural factors strongly influence reproductive patterns and affect the generalisability from modern industrialised, low-fertility populations to ancestral conditions. Simultaneously, it is important to acknowledge that women in modern-day high-fertility contexts can and sometimes do manage their fertility using non-pharmacological methods (Colleran & Mace, 2015). However, as we have previously noted regarding men’s masculine traits (Lidborg *et al*., 2022), if feminine traits have been associated with increased fertility *on average* across the diverse cultural and ecological contexts of human history, we would still expect to find evidence of these associations *on average* in present populations.

Perhaps the most striking conclusion from this review is how little empirical evidence there is assessing the associations between some of women’s morphological traits and fecundity. Arguments for such an association have existed in the human sexual selection literature since the 1970s; the first to link women’s morphological traits with their fertility was Symons (1979). Since then the ‘fertility’ hypothesis has become widespread in evolutionary discourse, and has persisted over the decades (see e.g. Bovet, 2019; Buss & Schmitt, 1993, 2019; Cunningham, 1986; Gottschall, 2007; Jasieńska *et al*., 2004; Johnston, 2000; Law Smith *et al*., 2006; Singh, 1993; Singh & Singh, 2011; Sugiyama, 2005). As discussed in the Introduction, however, this hypothesis – which does not necessarily lack merit – is not well tested when it comes to *actual* fertility rather than *indices* thereof (and incidental evidence should be treated with caution). Our review does not suggest that the fertility hypothesis is incorrect. Rather, we argue that our review calls for greater caution making this claim in the absence of empirical evidence, while encouraging further research in this area.

Nonetheless, this lack of evidence means that the frequency and fervour with which the fertility hypothesis is repeated in the literature is somewhat surprising. For example, we did not locate a single study measuring fertility as a function of facial femininity. Given that facial femininity and facial attractiveness are strongly correlated, at least in industrialized samples (Fiala *et al*., 2021; Perrett *et al*., 1994; Rhodes, 2006), they are often treated as measuring largely the same thing. However, as strongly noted by Scott *et al.* (2014), most facial dimorphism research is conducted in ‘WEIRD’ (western, educated, industrialized, rich, and democratic) samples, with evidence from traditional, small-scale societies not necessarily supporting the notion that facial femininity is always or universally attractive to men (e.g. Boothroyd *et al*., 2021). To treat attractiveness as a proxy for femininity in evolutionary arguments regarding our species as a whole is therefore inadvisable. As this review focused specifically on feminine traits, we chose not to include measures of facial attractiveness here. Overall, it remains an open question whether women’s facial shape bears any relationship with their fertility.

The fact that facial femininity continues to be described in the literature as a fertility/fecundity marker, due to a putative association with oestrogen, is further problematic given that the theoretical grounds for craniofacial femininity being an evolved signal or cue communicating fecundity are weak. First, as mentioned in the Introduction, the evidence for adult femininity reliably indexing hormone levels is not conclusive. (Of course, even when hormone levels and trait expression *are* correlated, such a correlation does not in itself constitute evidence that the trait is naturally or sexually selected; trait expression can also occur as a by-product of hormone exposure: Gould & Lewontin, 1979.) Second, unlike facial masculinity in men, facial femininity in women does not become exaggerated at sexual maturity and is thus technically not a secondary sexual characteristic. While the fuller lips seen in women compared to in men are often claimed to be oestrogen-dependent (e.g. Rhodes, 2006), the evidence for a relationship between oestrogen and other feminine facial traits is scarce. Combined with the fact that female faces cease to grow in puberty (Bulygina *et al*., 2006) – when oestrogen levels are high and stimulate exaggeration of other feminine traits such as enlarged breasts and a gluteofemoral fat distribution – and women thus retain relatively juvenile facial characteristics beyond adolescence and into adulthood, this suggests that a feminine craniofacial structure is not dependent on oestrogen. Rather, human facial dimorphism appears to largely result from testosterone exposure influencing the growth of men’s faces (Swaddle & Reierson, 2002), weakening the claim that facial femininity in women communicates reproductive potential. Combining this with the lack of empirical work testing whether facial femininity is associated with fertility calls for further research. In the meantime, we caution researchers against repeating hypotheses for which there is no extant evidence.

### 4.1. Limitations and future directions

Publication bias skewing the results is a common concern in systematic reviews. Ideally, this should be assessed, for example through graphical tools such as funnel plots with an accompanying Egger’s test (Page *et al*., 2024) or through *p*-curve analysis. Here, the small number of studies prevented us from employing such methods: the Cochrane Handbook advises that funnel plots should not be used for reviews that include fewer than 10 studies, which was the case for all our measures. *p*-curve analysis is similarly more reliable when a larger number of studies are available (Simonsohn *et al*., 2014). For the trait with the greatest number of significant effects here (2D:4D ratio), only seven significant effects were available, which was considered too few to conduct a meaningful *p*-curve analysis. The fact that we were not able to formally assess publication bias is therefore a limitation of the present review (indeed, the small number of studies found for the majority of measures also meant we were not able to conduct a formal meta-analysis).

We did, however, follow all other methods recommended by the Cochrane Handbook to reduce the risk of bias, wherever these were applicable. For example, we attempted to find unpublished data by making calls for data on social media; these attempts were unsuccessful. Further, where articles included variables of interest but where the relevant analyses were not provided, we contacted the authors asking them for the relevant effect sizes (an approach that was successful in identifying unpublished effects in our previous meta-analysis), but none of the contacted authors were able to provide them. Citation searches of all eligible studies on Google scholar ensured that unpublished work (e.g. dissertations, theses, and other ‘grey literature’) would have been found. We are therefore quite certain that we employed all means available to us to reduce the risk of bias. It should also be noted that the majority of published effects we found (59%) were nonsignificant. Thus, although we cannot completely rule out the possibility of publication bias, visual inspection of published effects does not strongly suggest it.

Furthermore, as noted in the Introduction, researchers have often posited that morphological traits are indicative of current hormone levels, despite the fact that these traits may more accurately reflect pubertal hormone levels. Combined with the fact that certain traits (such as breast appearance and WHR) are influenced by pregnancy and breastfeeding, future research should ideally use longitudinal designs to assess the association between both facial and body traits – measured in adolescence or at least pre-marriage/pre-reproduction – and lifetime fertility. Doing this would also enable researchers to determine whether the putative relationship between women’s morphology and fertility is mediated by age at/likelihood of marriage, which in itself is not a measure of potential hormone-mediated fecundity, but which is likely to influence lifetime fertility. Naturally, non-contracepting populations would be most suitable to sample from when testing these associations, although they should not be viewed as direct parallels to ancestral humans. Nonetheless, removing the complication of widespread, highly effective contraception use from data can give a stronger test not only of the hypothesis that feminine traits function as biological fecundity markers (which can be tested in any population), but that this fecundity results in more children for long-term partnered women (the fundamental mechanism supposed to underlie the evolution of the trait). We recognize that, although the most robust way to test these associations, this can be logistically and financially challenging. We therefore encourage researchers conducting cohort studies to include and report comprehensive measures that may be of interest across a range of research areas and to engage in cross-discipline collaborations. It is also possible that existing databases may contain relevant variables not yet analysed, which could be of great interest to test evolutionary-drawn hypotheses (e.g. several of the effects included here were drawn from the medical literature). This could potentially also address the sample size issue discussed previously.

### 4.2. Conclusion

In summary, we systematically reviewed the literature investigating whether women’s morphological traits predict fertility. The aim of this review was not to ascertain whether women’s feminine morphological traits evolved or are currently maintained through natural or sexual selection pressures, but to review extant evidence for this association in the first place. We found weak evidence that a more feminine 2D:4D was correlated with higher fertility, but effect sizes were small. A higher (i.e. less feminine) WHR was associated with greater past fertility, but had not been sufficiently tested prospectively for us to be able to assess its relationship with future fertility. Breast size, voice pitch, and strength/muscle mass had also been tested in too few studies for us to draw any firm conclusions. Unfortunately, not a single study had measured fertility as a function of the most prominently studied trait in female sexual selection research, namely facial femininity; we encourage evolutionary scholars to address this question in future research. Overall, we make the following concluding remarks. (i) Direct empirical evidence for the claim that women’s feminine traits are sexually selected and cue fertility is at present largely absent and we encourage wider acknowledgement in the evolutionary literature that this association has not yet been demonstrated. (ii) Further, we call for greater use of longitudinal studies incorporating direct measures of feminine morphological traits and actual fertility.

## Research transparency and reproducibility

A preprint version of this article was previously uploaded to PsyArXiv and is available at https://doi.org/10.31234/osf.io/nhtre.

This systematic review is based on publicly available data from previously published articles. All data sources are cited, and no new primary data were generated or analysed for this review. One effect size (Wells *et al*., 2010) was calculated for this systematic review based on information available in the relevant article. Readers can access the data through the referenced publications and repositories.
